# Cross-sectional area variations of internal jugular veins during supine head rotation in multiple sclerosis patients with chronic cerebrospinal venous insufficiency: a prospective diagnostic controlled study with duplex ultrasound investigation

**DOI:** 10.1186/1471-2377-13-162

**Published:** 2013-11-05

**Authors:** Massimiliano Farina, Eugenio Novelli, Raffaello Pagani

**Affiliations:** 1 Phlebolymphology Diseases Centre, Monza Polyclinic, Monza, Italy; 2 Biostatistics Unit, San Gaudenzio Clinic (Monza Polyclinic Group), Novara, Italy; 3 Angiology Unit, Villa Cimarosa Medical Centre, Milan, Italy

**Keywords:** CCSVI, MS, Jugular wall miopragia, Hourglass appearance, SCM stretching manoeuvre, Balloon angioplasty, Type I/III collagen

## Abstract

**Background:**

Normally, chronic cerebrospinal venous insufficiency (CCSVI) has been studied using echo-colour Doppler (ECD). Subjects are examined in the supine and sitting positions, in accordance with a static protocol without rotation of the head. A dynamic approach, to assess venous sizes with different degrees of head rotation, has only been performed to improve jugular venous catheterisation. These echographic studies have suggested that head rotation to the contralateral side increases the cross-sectional area (CSA) of the internal jugular veins (IJVs) in supine subjects. Our goal was to evaluate the behaviour of CSA of the IJVs during supine head rotation in multiple sclerosis (MS) patients with CCSVI, compared to healthy controls (HCs).

**Methods:**

The IJVs of 313 MS patients with CCSVI (male 43.8%, male/female 137/176; mean age 45 years old, range 19–77 years) and 298 HCs, matched by gender (male 43.6%, male/female 130/168) and age (mean age 46 years old, range 20–79 years), were compared using ECD. Their CSAs were evaluated with the subjects seated in a tiltable chair, first in the supine position at the level of the cricoid cartilage, with the head in a neutral position, and then after contralateral rotation to 90° from midline.

**Results:**

Significant differences between the jugular CSAs before and after head rotation were observed only in the MS patients for the IJVs with wall collapse (F[6,1215] = 6414.57, p < 0.001), showing on longitudinal scans a typical “hourglass” aspect that we defined as “miopragic”. No significant difference was found in the distribution of these miopragic veins with regard to MS duration. There was a strong association between the CCSVI scores and the complexity of jugular morphological types (*Χ*^2^ [9, N = 313] = 75.183, p < 0.001). Wall miopragia was mainly observed in MS patients with SP (59.3%) and PP (70.0%) clinical forms, compared to RR (48.3%) forms (p = 0.015).

**Conclusion:**

A dynamic ECD approach allowed us to detect IJVs with a significant increase in their CSAs during head rotation, but only in MS subjects. This feature, most likely the expression of congenital wall miopragia, could be secondary to dysregulation of collagen synthesis, but further histochemical studies will be needed to confirm this hypothesis.

## Background

Chronic cerebrospinal venous insufficiency (CCSVI) is a congenital syndrome affecting the extracranial vessels (internal jugular and azygos veins) that is characterised by different valve malformations, stenoses and segmental or global hypoplasia with impaired venous drainage, opening collateral circulation [1-3]. Several studies have demonstrated a strong association between multiple sclerosis (MS) and CCSVI, but subsequent clinical research has failed to support this hypothesis [4-13]. Thus, the debate currently continues over whether the relationship is real. In fact, some reports have claimed that the data in the literature have been insufficient to establish the importance of CCSVI as a major factor in MS pathogenesis. Echo-colour Doppler (ECD) studies of CCSVI are usually performed according to the five Zamboni criteria; the detection of at least two of these parameters indicates a diagnosis of CCSVI. Nevertheless, ECD investigation is still not currently standardised in many respects, and it is overly dependent on individual patients and operators, with high interobserver variability for untrained examiners [14,15]. However, different studies have reported that ECD is more sensitive than the magnetic resonance venography (MRV) in detecting intraluminal jugular defects, while MRV is more sensitive in showing collaterals [9,16]. Both techniques have proved effective in assessing the size and course of venous vessels of the neck, showing internal jugular vein (IJV) asymmetry in both normal subjects and MS patients. The left internal jugular vein is usually smaller than the right due to preferential intracranial venous drainage through one sigmoid sinus versus the other, with weak correlations with age and gender [17-21]. Venous size can vary depending on hydration status, position, cardiac status, thoracic pump, head position, and compression from adjacent structures [22-26]. Even dysfunction of the cardiovascular autonomic nervous system can reduce vascular tone, affecting jugular size [27]. More frequently, MS patients have shown a jugular cross-sectional area (CSA) ≤30 mm^2^ compared to healthy controls (HCs) (43.5% vs. 16.7%) [28]. These ultrasonographic findings were also confirmed by catheter venography (CV) and were mainly detected in the middle part of the IJV, at the level of the cricoid cartilage immediately below the sternocleidomastoid muscle (SCM) [29]. They have not been clearly correlated with intraluminal defects, although they have been explained by low values of internal pressure [30]. At this level, CV and ECD have shown a collapsed jugular vein with loss of its elliptical appearance under SCM imprinting [5,29]. Patients have usually been examined in the supine and sitting positions, in accordance with a static protocol without head rotation (0° from midline). A dynamic approach to assess venous sizes with different degrees of head rotation has only been applied to improve jugular venous catheterisation. These echographic studies have suggested that head rotation to the contralateral side increases the CSA of IJVs in supine subjects [25,31]. The aim of this study was to evaluate the behaviour of the CSA of the IJVs during supine head rotation in MS patients with CCSVI, compared to HCs.

## Methods

### Patients and controls

Between June 2010 and November 2012, we studied 313 patients with clinically defined MS (according to the 2010 revised McDonald diagnostic criteria) and CCSVI, who were diagnosed with the presence of at least two of the five Zamboni criteria [1,32]. This group consisted of 172 patients with the relapsing-remitting (RR) clinical form, 91 with the secondary progressive (SP) form and 50 with the primary progressive (PP) form. An MS specialist evaluated all the patients, assigning disability scores according to the Kurtzke scale (EDSS). Table 1 shows the related demographic and clinical characteristics. We also studied 298 volunteers HCs (students and technical and administrative staff from our hospital) with a mean weight of 70.23 kg (standard deviation [SD] = 7.97; range: 43–98), who were matched by gender (male 43.6%, male/female 130/168) and age (mean age 46 years old, range 20–79 years). Physical and neurological examinations were performed on the controls. We excluded from the study any subject with previous head or neck surgery, neck swellings, severe heart disease, serious kidney and liver diseases, thrombosis of the jugular vein(s), jugular vein catheterisation, vasculitis, Behçet’s syndrome, collagen diseases, congenital cerebral malformations and congenital vascular malformations. The Ethical Committee of the Local Health Authority, Monza Brianza, Italy, approved this prospective, diagnostic, controlled study, and all the participants provided written informed consent.

**Table 1:** Demographic and clinical characteristics of MS patients with CCSVI

	**Whole MS**	**RR form**	**SP form**	**PP form**
	**(N = 313)**	**(N = 172)**	**(N = 91)**	**(N = 50)**
Age (years) (Mean [range])	45 (19–77)	39 (19–69)	50 (32–77)	54 (21–76)
Gender (% male -n male/female)	43.8 (137/176)	40.1 (69/103)	43.9 (40/51)	56 (28/22)
Body weight (kg) (Mean ± SD) (range)	67.47 ± 7.12 (49–88)	67.40 ± 6.55 (51–84)	67.49 ± 7.54 (49–82)	67.68 ± 8.28 (52–88)
EDSS (Mean [range])	3.5 (1–9)	3.0 (1–8)	5.0 (2–9)	4.0 (1–8)
Disease duration (years) (Mean [range])	11 (1–40)	7 (1–30)	15 (1–38)	14 (1–40)

### Duplex ultrasound investigation

#### Classic approach

The classic approach was applied as proposed in 2011, according to the revised protocol by an expert panel of the International Society for Neurovascular Disease (ISNVD) [33]. A single experienced vascular sonographer performed all the investigations in the morning, with the subjects first placed in the supine position (0°) and then in the upright sitting (90°) position. An ECD system (MyLabVinco, Esaote SpA, Florence, Italy), equipped with a linear array transducer probe with an operating bandwidth of 3–11 MHz (B-modes frequencies, 3.5 - 5.0 - 6.6 - 10.0 MHz; Doppler frequencies, 3.3 - 5.0 MHz) for extracranial scans, was used. The transcranial approach was performed with a phased array transducer probe with an operating bandwidth of 1–4 MHz (B-modes frequencies, 2.0 - 2.5 - 3.3 MHz; Doppler frequencies, 1.6 - 2.0 - 2.5 MHz). The vascular sonographer was particularly experienced with venous disease and had performed approximately 10,000 ultrasound investigations per year over the past few years. Each subject underwent an examination of laterocervical area of the neck, exploring both the internal jugular and vertebral veins. We also evaluated deep cerebral veins from the transcondylar window. Longitudinal supine scans of the IJVs were obtained from the distal part (J3) above the carotid bifurcation to the subclavian junction (J1), passing through the intermediate portion at the level of the cricoid cartilage (J2). All the scans were recorded for subsequent reconstruction and morphological analysis. The longitudinal diameters were measured at the levels of J1, J2 and J3. Measurements of the cross-sectional area (CSA - mm^2^) of the IJVs were obtained in real-time at the same point (J2), by placing the transducer probe over the apex of the clavicle-sternocleidomastoid triangle at the level of the cricoid cartilage and perpendicular to the skin. For the computations, we used an ellipsoid or continuous trace method, referring to the greatest ellipse at the end-expiratory phase. Each supine measurement (longitudinal diameters and CSAs) was repeated three times, and the average of the three measurements was used for comparison. These basal assessments were performed with the subject’s head in a neutral position (0° midline), which was defined as having the subject’s sagittal plane perpendicular to the supporting surface. In the supine position, a small pillow (8 cm height) was placed under the subject’s head to induce a greater degree of relaxation of the neck musculature [34]. On the examined skin, we used a large amount of gel to assure perfect coupling of the transducer, to reduce excessive pressure, and to avoid changing the IJV shape and dimension. During the position changes, each subject was kept in a resting condition (no voluntary muscle movements and contractions) as much as possible, using a proper electromechanical tiltable chair. Adequate fluid intake was maintained during the 24 hours preceding the survey (500 ml upon waking before the exam) to avoid dehydration, which can affect morphological detection of the IJVs.

#### Dynamic approach: the sternocleidomastoid muscle-stretching manoeuvre

**Figure 1:**
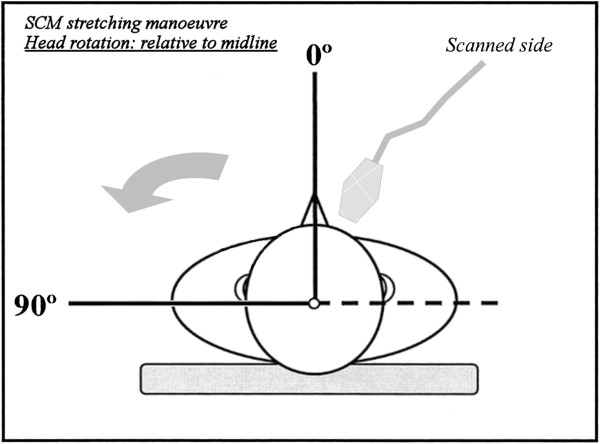
Maximum contralateral head rotation compared to the scanned side.

After basal evaluations, each supine subject underwent a dynamic manoeuvre that consisted of contralateral rotation of the head to 90° from midline compared to the scanned side, with the chin slightly raised (Figure 1). In this manner, we obtained the elongation and thinning of the SCM, with external pressure reduction on the middle portion of the jugular vein and a related increase in the venous section, as reported in normal subjects by several authors in studies to optimise central venous cannulation [20,25]. At the same level, we also obtained a second measurement of the CSA of the IJVs during the end-expiratory phase. This measurement was the result of the average of three consecutive detections. Subsequently, we calculated the difference between the mean values of the jugular CSAs in both positions (ΔCSA), by subtracting the area obtained in the neutral position from that obtained in maximum contralateral rotation.

#### IJV morphological types

**Figure 2:**
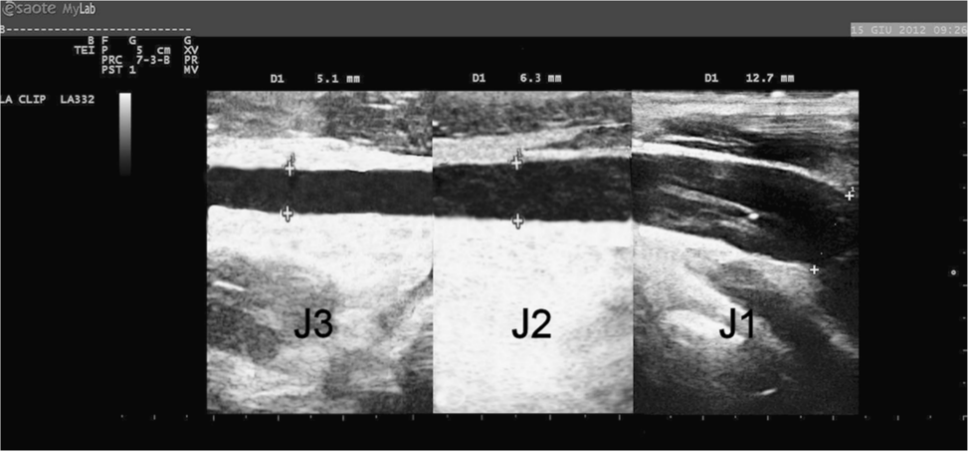
**A normal IJV.** Progressive reduction of the vessel diameter proceeding from J1 point (12.7 mm) to J3 (5.1 mm) and passing through J2 (6.3 mm).

**Figure 3:**
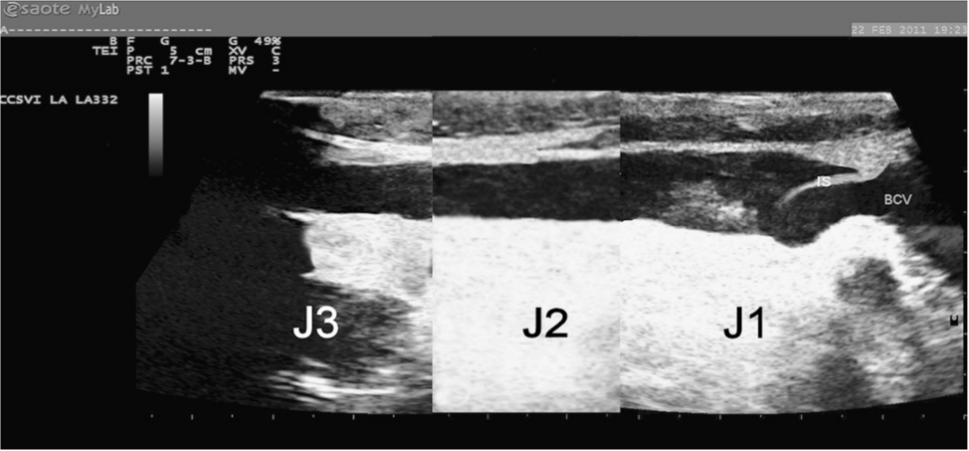
**Proximal (J1) valve malformation (inverted septum).** BCV = brachiocephalic vein.

**Figure 4:**
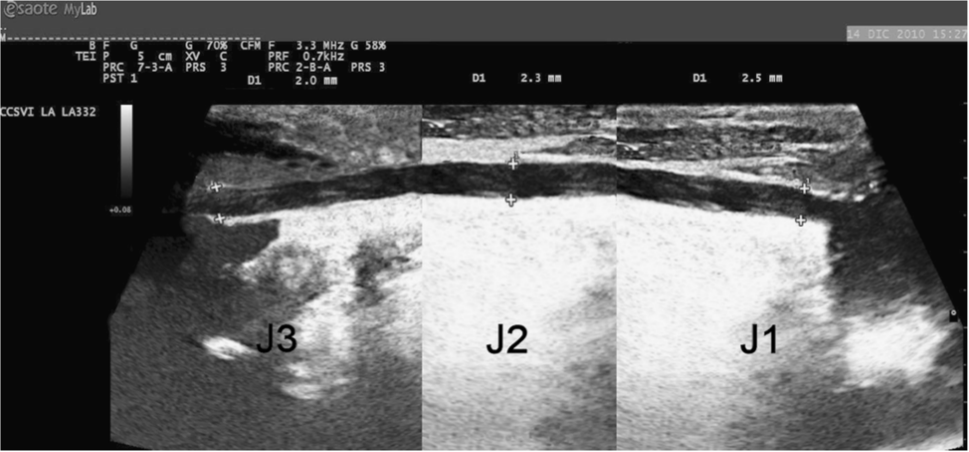
**An hypoplastic IJV (cylindrical appearance).** The diameter (between 2.0 and 2.5 mm) is broadly similar for all lengths.

A careful analysis of the literature allowed for the identification of three different morphological types of IJVs: normal veins; veins with only VDs; and hypoplastic vein (small veins). The reconstruction of the longitudinal ultrasound scans of the IJVs provided a better definition and understanding of these morphological types. A normal jugular vein gradually reduces its longitudinal diameter passing from J3 to J1, as described in several texts on human anatomy [35] and as recently confirmed by CT [36] and ultrasound studies [19,20]. Therefore, the vein presents a typical “telescopic” appearance (Figure 2). More recently, studies involving MS patients have revealed the presence of IJVs with intraluminal defects, such as proximal malformed valves and septa webs (Figure 3) [1,4,5]. A hypoplastic jugular vein is much smaller. A recent CT study [36] defined as hypoplastic those IJVs with proximal longitudinal anteroposterior diameters (J1) two SDs less than the mean (≤5.45 mm). With regard to the area, these veins showed a CSA ≤30 mm^2^[33]. In these forms, the vessels appear to be “cylindrical”, with the diameter broadly across the entire length (Figure 4).

### Statistical analysis

Continuous variables are described as means and standard deviations (SDs), while counts and percentages are used to describe qualitative variables. A frequency matching approach for gender and age was applied in this study. On the basis of the distribution quartiles, four classes of disease duration (<5, 5–10, 11–16, >16 years) were used to test the hypothesis that miopragia is congenital. Differences in ΔCSA measurements, before/after the manoeuvre in patients and controls, among the IJV morphological types are reported with means ± SDs and were analysed using a one-way analysis of variance (per IJV analysis).

The association between the complexity of the IJV morphological types of the patients and their CCSVI scores was tested using *X*^2^ statistics (per patient analysis). Kruskal-Wallis one-way analysis of variance was used to determine the explanatory power of the new diagnostic classification of IJV malformations in CCSVI scoring and to test whether the CCSVI score differed among the groups of patients, which were based on IJV morphological types. Post-hoc pairwise comparisons after Kruskal-Wallis analysis were performed with the Mann–Whitney U test and with p-values adjusted for multiple comparisons, which were performed using the Holm-Bonferroni method. The statistical analyses were performed using SPSS software (version 14.0, SPSS Inc., Chicago, IL, USA). All 2-tailed p-values less than 0.05 were considered statistically significant.

## Results

**Figure 5:**
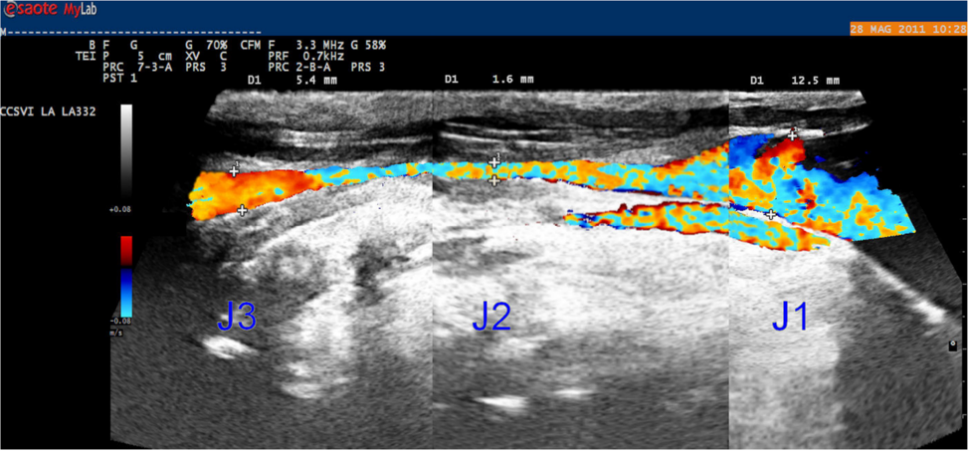
**IJV with wall miopragia (hourglass appearance).** Narrowing of the middle segment (J2 - 1.6 mm) with larger values at each end (12.5 and 5.4 mm at J1 and J3 points, respectively).

**Figure 6:**
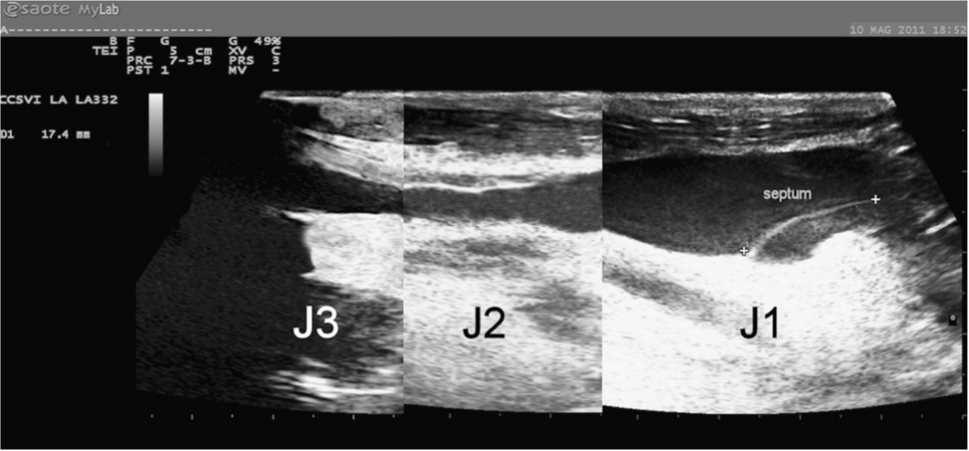
A miopragic IJV with proximal valve malformation (posterior septum).

**Figure 7:**
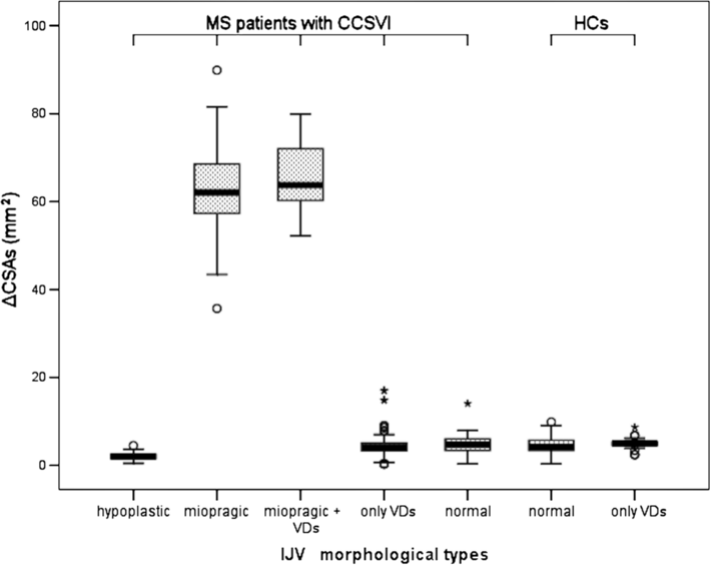
**The ΔCSA measurements after head rotation in MS patients and HCs.** Differences with respect to their IJV morphological types (per IJV analysis).

A total of 1,222 IJVs were evaluated: 596 in the HCs group and 626 in the MS group. Table 2 shows the distribution of the five Zamboni criteria in the two groups. As reported in the literature, our reconstruction of the longitudinal ultrasound scans of IJVs in MS patients detected normal veins (11.7%), veins with only VDs (49.2%) and hypoplastic veins (7.5%). We also identified two new morphological types of IJVs, which we defined as “miopragic” (27.1%) and “miopragic with VDs” (4.5%). A miopragic jugular vein has a typical aspect of an “hourglass”, with an extremely small diameter in the intermediate portion (mean ± SD = 3.0 ± 0.8 mm) and larger values at the J1 (10.5 ± 1.0 mm) and J3 levels (5.3 ± 0.6 mm) (Figure 5). In 4.5% of cases, miopragic veins presented with proximal VDs and mean diameter values that were comparable to previous types (J1 = 10.2 ± 1.1 mm; J2 = 3.3 ± 0.7 mm; J3 = 5.2 ± 0.5 mm) (Figure 6). The HCs showed normal jugular veins and proximal VDs in 93.5% and 6.5% of cases, respectively. In addition, we detected no miopragic or hypoplastic types in this subject group. Table 3 clearly describes these results, and as shown, the differences in frequency of IJV morphological types between the patients and controls were highly significant (*Χ*^2^ [4, N = 1222] = 825.129, p < 0.001). In a per IJV analysis, we compared the ΔCSA measurements after head rotation in the MS patients and HCs, with regard to their IJV morphological types. Significant differences between jugular CSAs before and after the reported manoeuvre were only observed in the MS patients (*F*[6,1215] = 6414.57, p < 0.001). The results are summarised in Table 4 and Figure 7. In a per patient analysis, we also correlated the IJV morphological types with the CCSVI scores (Table 5). A strong association was observed between the IJV morphological types and the CCSVI scores (*Χ*^2^ [9, N = 313] = 75.183, p < 0.001). In fact, the patients with isolated VDs had a median CCSVI score of two, those with hypoplasia plus VDs or miopragia plus VDs had a median score of three, and the patients with hypoplasia of an IJV and miopragia plus VDs of the other IJV had a median score of four. The above-mentioned classification of the complexity of the patients’ IJV morphological types was able to explain the variability of CCSVI scores in the MS group. In our series, we reported a statistically significant difference in the mean ranks of CCSVI scores among the different morphological types of IJVs (Kruskal-Wallis H[3] = 48.094, p < 0.001). The mean rank was 110.60 for isolated VDs, 153.59 for hypoplasia plus VDs, 177.93 for miopragia plus VDs and 219.43 for patients with hypoplasia of an IJV and miopragia plus VDs of the other IJV. Table 6 shows post-hoc paired comparisons of CCSVI scores among the IJV morphological types after Kruskal-Wallis analysis. The patients with hypoplasia + miopragia + VDs had higher CCSVI scores than patients with hypoplasia + VDs (H_3_ hypothesis, p = 0.033), while the patients with hypoplasia + VDs had higher CCSVI scores than patients with only VDs (H_4_ hypothesis, p = 0.024). Likewise, the patients with miopragia + VDs had higher CCSVI scores than patients with only VDs (H_5_ hypothesis, p < 0.001), and the patients with hypoplasia + miopragia + VDs had higher CCSVI scores than patients with only VDs (H_6_ hypothesis, p < 0.001). Wall miopragia was mainly observed in MS patients with the PP (70.0%) and SP (59.3%) clinical forms compared to the RR (48.3%) form (p = 0.015). In contrast, no significant differences in the observed frequencies of wall miopragia were noted among the four classes of disease duration (56.0%, 58.0%, 45.3%, 60.6%; p = 0.260); this result did not change even after distinguishing for clinical forms.

**Table 2:** Five Zamboni criteria distributed in both subject groups

**Colour Doppler criteria**	**Relapsing remitting**	**Secondary progressive**	**Primary progressive**	**Whole MS**	**Healthy controls**
	**(N, %)**	**(N, %)**	**(N, %)**	**(N, %)**	**(N, %)**
1. Reflux in IJVs and/or VVs with the head at 0° and +90° or reflux in one position with blocked flow in the other	91/313	58/313	29/313	178/313	18/298
	29.1%	18.5%	9.3%	56.9%	6.0%
2. Reflux in the deep cerebral veins	162/313	88/313	49/313	299/313	64/298
	51.8%	22.1%	15.6%	95.5%	21.5%
3. High resolution B-mode evidence of proximal IJV malformations	162/313	90/313	42/313	294/313	39/298
	51.8%	28.8%	13.3%	93.9%	13.1%
4. Flow not Doppler detectable in the IJV and/or VV at 0° and 90°	22/313	28/313	18/313	68/313	0/298
	7.0%	8.9%	5.7%	21.7%	0%
5. ΔCSA in the IJV ≤ 0	20/313	29/313	13/313	62/313	0/298
	6.4%	9.3%	4.1%	19.8%	0%

**Table 3:** Distribution of IJV morphological types in both subject groups (per IJV analysis, N = 1222)

	**MS patients with CCSVI**	**Healthy controls**
1. Normal	73/626 11.7%	557/596 93.5%
2. Only VDs	308/626 49.2%	39/596 6.5%
3. Hypoplasia	47/626 7.5%	0/596 0%
4. Miopragia	170/626 27.1%	0/596 0%
5. Miopragia + VDs	28/626 4.5%	0/596 0%

**Table 4:** Cross-sectional area variations (ΔCSA) of IJVs after head rotations (per IJV analysis, N = 1222)

		**Right or left CSA (mm**^**2**^**) (Mean ± SD)**		**ΔCSA (mm**^**2**^**) (Mean ± SD)**		**Right and left CSA (mm**^**2**^**) (Mean ± SD)**		**ΔCSA (mm**^**2**^**) (Mean ± SD)**
**IJV morphological types**	**N**	**Neutral position (0° - midline)**	**Max. rotation (90° from midline)**		**N**	**Neutral position (0° - midline)**	**Max. rotation (90° from midline)**	
Normal	73	69.685 ± 8.763	74.395 ± 9.052	4.710 ± 1.987	557	70.084 ± 9.098	74.613 ± 9.067	4.529 ± 1.675
Only VDs	308	71.050 ± 8.876	75.461 ± 8.746	4.411 ± 2.079	39	71.431 ± 7.943	76.374 ± 8.549	4.944 ± 1.168
Miopragic	170	9.921 ± 2.554	72.695 ± 8.413	62.775 ± 8.940 (*)	0	-	-	-
Miopragic + VDs	28	8.682 ± 1.647	74.400 ± 6.483	65.718 ± 7.070 (*)	0	-	-	-
Hypoplastic	47	9.096 ± 2.443	11.132 ± 2.414	2.036 ± 0.844	0	-	-	-
	*MS patients with CCSVI*				*Healthy Controls*			

* p < 0.001.

**Table 5:** Distribution of IJV morphological types related to CCSVI score (per patient analysis, N = 313)

	**CCSVI score**			
	**2**	**3**	**4**	**5**
	**(N, %)**	**(N, %)**	**(N, %)**	**(N, %)**
1. Only VDs	58/94	28/94	8/94	0/94
	61.7%	29.8%	8.5%	0%
2. Hypoplasia + VDs	11/33	17/33	5/33	0/33
	33.3%	51.5%	15.2%	0%
3. Miopragia + VDs	34/172	103/172	24/172	11/172
	19.8%	59.9%	14.0%	6.4%
4. Hypoplasia + Miopragia + VDs	2/14	4/14	8/14	0/14
	14.3%	28.6%	57.1%	0%

**Table 6:** Post-hoc paired comparisons after Kruskal-Wallis analysis

**Hypothesis**	**Observed**	**Holm threshold**	**Holm adjusted p-value**
	**p-value**	**value**	**=max{6p**_**6**_**, 5p**_**5**_**, 4p**_**4**_**, 3p**_**3**_**, 2p**_**2**_**, p**_**1**_**; kp**_**k**_**}**
			**k = 1, 2, 3, 4, 5, 6**
H_1_	0.102	stop	0.102 = max (0, 0, 0.024, 0.033, 0.070, 0.102)
H_2_	0.035	0.05/2 = 0.025	0.070 = max (0, 0, 0.024, 0.033, 0.070)
H_3_	0.011	0.05/3 = 0.017	0.033^ = max (0, 0, 0.024, 0.033)
H_4_	0.006	0.05/4 = 0.013	0.024^ = max (0, 0, 0.024)
H_5_	0.000	0.05/5 = 0.010	0.000^ = max (0, 0)
H_6_	0.000	0.05/6 = 0.008	0.000^ = max (0)

H_1_: Hypoplasia+VDs *vs.* Miopragia+VDs, H_2_: Miopragia+VDs *vs.* Hypoplasia+Miopragia+VDs.

H_3_: Hypoplasia+VDs *vs.* Hypoplasia+Miopragia+VDs, H_4_: Hypoplasia+VDs *vs.* VDs, H_5_: Miopragia+VDs *vs.* VDs.

H_6_: Hypoplasia+Miopragia+VDs *vs.* VDs. The hypotheses have been sorted in descending order of the observed p-values.

^: statistically significant result.

## Discussion

**Figure 8:**
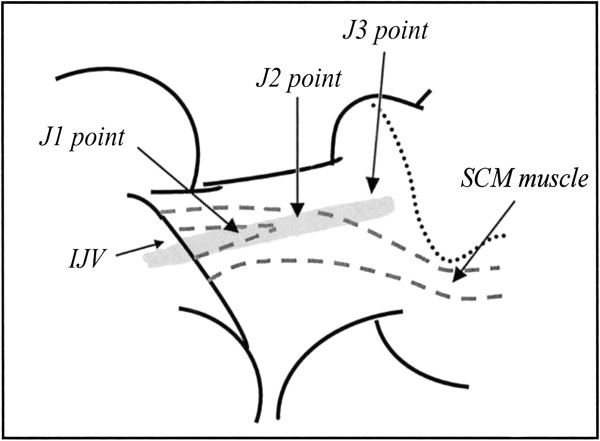
Anatomical relationships between SCM and IJV segments.

Currently, there is much ECD evidence regarding the presence of proximal jugular valve malformations with altered local flow in MS patients with different prevalence data according to the authors [1,7,15,37]. In our experience we have found CCSVI in 89.8% of MS patients and in 5.4% of HCs. Similar results have been obtained with other diagnostic imaging techniques, such as MRV [9,38,39] and CV [8,37]. However, all diagnostic methods have technical issues, and not all are completely standardised [9,14]. Since 2009, jugular abnormalities have been included in the Consensus Document of the International Union of Phlebology (IUP) on venous malformations and have been classified as congenital truncular lesions [40], but until now, little was known regarding their histopathogenesis. The recent work of Coen was the only study that addressed the issue from this point of view, and it was the first that detected an altered ratio of type I/III collagen in the IJVs of MS patients, without any differences in cellularity or connective tissue distribution [41]. This condition has been similarly described in many other apparently unrelated conditions (e.g., varicose saphenous veins, haemorrhoids, paraoesophageal hernia, skin incisional hernia, recurring inguinal hernia, pelvic organ prolapse), suggesting connective tissue systemic involvement [42-44]. These structural changes were also found in vessel wall samples taken far from balloon angioplasty treatment areas, suggesting their independence on local trauma. As evidenced by several studies, type I collagen provides mainly tensile strength and rigidity by building thick fibre that offer resistance to tissue, while type III collagen determines elasticity [43]. These histochemical data could explain the typical hourglass appearance of a part of the IJVs that we defined as “miopragic”. This morphological type was only detected in MS patients in the supine neutral position, with related flow blocked in their middle portions. It could be the result of vein collapse under SCM pressure because of reduced wall stiffness (wall miopragia). In fact, in the resting position, muscle pressure is greater at the J2 level, where the SCM is larger and closer to the IJV, crossing it to pass medially. Here, the vein is usually positioned anterolateral to the carotid artery [45]. Similarly, external pressure (exerted on the vein) is less in the distal segment (J3), where the SCM is located lateral to the vessel, and also in the proximal portion (J1), where the jugular passes among the sternal and clavicular heads of the same muscle (Figure 8). In addition, the regular outflow along the IJV ensures its patency at the J3 level, with a shunt through the common facial vein towards the external or anterior jugular veins. The inferior thyroid veins mainly support J1 outflow. Contralateral rotation of the head restores regular flow along the vein through the significant increase (*F*[6,1215] = 6414.57, p < 0.001) in CSA in the intermediate section, which was only observed for this morphological type (see Additional files 1, 2 and 3) and was never detected in the HCs. This result in the HCs agrees with what Lorchirachoonkul and Suarez found in normal subjects, namely a non-significant increase in jugular CSAs and longitudinal diameters with contralateral rotation of the head [19,25]. However, the jugular collapse could also be an expression of cardiovascular autonomic disorders, as described in MS patients by several authors [27,46]. A dysregulation of vegetative function would reduce venous tone, causing jugular wall collapse under SCM pressure. However, this datum is controversial because older studies, as well as recent publications, have observed no anomalies on orthostatic tests [47,48]. According to other researchers, autonomic disorders are present in up to 23% of MS patients [49], and this finding would not explain the jugular collapse that we found in a greater number of subjects (54.9%). Among other findings, our data revealed no statistically significant difference in the mean CSA of normal IJVs between MS subjects with CCSVI and HCs in the supine position, as shown in another study [28]. Similarly, the distribution of mean values of the CSA of normal jugular veins in HCs in the supine position corresponded to what has been reported by several authors [25,28,50], with a trend toward an increase when passing from J3 to J1 [20,24] and with rotation of the head [19,25]. Table 7 summarises these results, and as shown, our values of CSA are similar to those of other researchers, while they differ from those of Zamboni’s two studies. We believe that our higher values might be due to the younger mean age and likely lower body weight of the participants in the Zamboni’s study, compared to our and other studies. In fact, as proved by Mortensen, the internal jugular size has the best positive correlation with body weight [21]. As reported in a recent work, the failure of jugular angioplasty in one MS patient with CCSVI showed a totally collapsed middle part of the vein [26]. Surgical decompression of an atypical omohyoid muscle successfully solved this case. Others have described the omohyoid muscle as involved in the physiologic dilatation of the IJVs during opening of the mouth and deep inspiration, through short segmentary compression of the proximal part of the vein (J1) near the tendinous intersection [51]. This physiological status could become pathological in cases of prolonged compression, as detected in the lower part of the neck, or in the presence of a shorter omohyoid directly merged with the sternohyoid muscle. Patra instead was not able to locate precisely the position of the omohyoid muscle and to measure the jugular surface below it because of poor muscle echogenicity [52]. However, anatomic variations in the inferior belly of the omohyoid muscle have been rare, seen in only 3% of autopsy findings [53]. In our study, all extrinsic jugular compressions occurred in the middle part (J2) of the vein below the SCM and were therefore not attributable to the omohyoid muscle action. When investigating MS patients using MRV, some authors have found extrinsic severe stenoses in 22% of the patients’ IJVs [16]. These findings indicate that CCSVI is mainly caused by valvular malformations [1,37] and that extrinsic compression by a muscle could represent a less frequent nonvalvular cause of compromised cerebral venous outflow [26,54]. We believe that jugular collapse, secondary to extrinsic muscular compression, is the expression of a congenital weakness of vessel walls, likely because of a dysregulation of collagen synthesis. We support our assertions with the presence of miopragic jugular veins (combined with VDs) in 54.9% of MS patients and the absence of them in the HCs, for whom a low percentage of VD (6.5%) was detected instead. Therefore, if jugular valve defects can also be recognised in HCs, wall miopragia would seem to be an exclusive feature of MS patients. If we add to this percentage the 10.5% of patients with hypoplastic jugular veins and the 4.5% of patients with combined malformations (hypoplasia + miopragia + VDs), the total value of MS patients with wall miopragia and VDs reaches 69.9%, compared with 30.1% of those with VDs only. These data assume greater importance, given the close correlation of vein miopragia to the degree of haemodynamic impairment of cerebrospinal efferent vessels. In fact, as noted in our per patient analysis, jugular miopragia was mainly correlated with medium to severe scores (3–4) for CCSVI, while patients with isolated VDs show lower scores (2). Similarly, combined malformations are correlated with higher CCSVI scores. We stated that wall miopragia would not affect the totality of MS patients because in our study, we found jugular veins with isolated VDs in 30% of subjects, but this number could be higher than reported. In fact, the collagen distribution might not be the same from one patient to another or even between different segments of the same vein; therefore, we could have jugular vessels with altered collagen but not sufficiently altered to induce wall collapse. These veins would appear as normal vessels with isolated VDs. Moreover, our data demonstrated a greater prevalence of jugular wall miopragia in patients with the PP and SP clinical forms, compared to the RR form (p = 0.015), and no significant relationship between jugular wall miopragia and MS duration, supporting the hypothesis that this morphological type is congenital. The principal limit of this study was the inability to confirm our hypothesis of jugular wall miopragia by means of a direct histochemical analysis of the vessels.

**Table 7:** Cross-sectional areas (CSAs) and anteroposterior (AP) diameters of IJVs in supine normal subjects with head in neutral position and after rotation

**Internal Jugular Veins (IJVs)**	**CSA (mm**^**2**^**)**		**Right CSA (mm**^**2**^**)**	**Right CSA (mm**^**2**^**)**		**CSA (mm**^**2**^**)**		
**Segment**	**Right 0°**	**Left 0°**	**20° contralateral rotation**	**0°**	**Max. contralateral rotation**	**Right and left 0°**	**Right 0°**	**Left 0°**
J1 (Mean ± SD)	-		-	-	-	17.8 ± 10.9	48 ± 30	50 ± 14
J2 (Mean ± SD)	84 ± 39	65 ± 31	9.0 ± 5.6	78 ± 46	111 ± 60	10.4 ± 8.4	37 ± 31	28 ± 19
J3 (Mean ± SD)	-		-	-	-	14.8 ± 13.2	26 ± 14	21 ± 11
N of IJVs	45	45	52		24	66	10	10
Body weight (kg) (Mean ± SD)	-		75.3 ± 15.8		-	-		
(range)	-		-		63-100.5	-		
Age (years) (Mean ± SD)	36.9 ± 11.8		37 ± 11		-	27.5 ± 5.0	-	
(range)	-		-		30-86	-	23-42	
Authors	*Kantarci et al.* [28]		*Bellazzini et al.* [50]		*Suarez et al.* [25]	*Zamboni et al.* [24]	*Zamboni et al.* [20]	
**Internal Jugular Veins (IJVs)**	**AP diameter (mm)**							
**Segment**	**Right 0°**		**Right 60°**		**Left 0°**		**Left 60°**	
J2 (Mean ± SD)	8.5 ± 2.7		9.1 ± 2.8		7.8 ± 2.7		8.2 ± 2.8	
N of IJVs	100				100			
Body weight (kg) (Mean)	65.6							
(range)	32–108							
Age (years) (Mean)	55.7							
(range)	21–89							
Authors	*Lorchirachoonkul et al.* [19]							

## Conclusions

The reconstruction of longitudinal echographic scans of IJVs provides a full view of vessels, thereby enabling the identification of a new morphological type with an hourglass appearance, which was not detected in the HCs. This type of vein shows a unique behaviour with the SCM stretching manoeuvre, most likely because of a condition of wall miopragia, the congenital nature of which was clearly shown by our study, but the meaning of which will have to be investigated further. This dynamic approach, applied to the conventional static ultrasound screening for CCSVI, allowed us to introduce the first selective criterion for angioplasty. In fact, it would be unthinkable to treat miopragic veins; balloon angioplasty would most likely fail because of the increased distensibility of the venous wall. Obviously, further histochemical studies will be needed to confirm whether jugular collapse, found in MS patients, could be the expression of dysregulation of collagen synthesis.

## Abbreviations

CCSVI: Chronic cerebrospinal venous insufficiency; CSA: Cross-sectional area; ECD: Echo-colour Doppler; HCs: Healthy controls; IJVs: Internal jugular veins; MRV: Magnetic resonance venography; PP: Primary progressive; RR: Relapsing-remitting; SCM: Sternocleidomastoid muscle; SP: Secondary progressive; VD: Valvular defect.

## Competing interests

The authors declare that they have no competing interests.

## Authors’ contributions

MF conceived of the study, participated in its design and coordination and primarily drafted the manuscript. EN participated in the design of the study and performed the statistical analysis. RP helped to draft the manuscript. All the authors read and approved the final manuscript.

## Pre-publication history

The pre-publication history for this paper can be accessed here:

http://www.biomedcentral.com/1471-2377/13/162/prepub

## Supplementary Material

Additional file 1 **SCM stretching manoeuvre.** B-mode transverse scan of a normal IJV at J2 level: a slight increase in jugular CSA with contra-lateral rotation of the head. Click here for file

Additional file 2 **SCM stretching manoeuvre.** Color-mode transverse scan of a miopragic IJV at the J2 level: the vein is not clearly visible in the frontal resting position (0° midline) and significantly increases its CSA with the opposite rotation of the head (90° from midline). Click here for file

Additional file 3 **SCM stretching manoeuvre.** B-mode longitudinal scan of a hypoplastic IJV at J1 level: there was an imperceptible change in the dimensional values observed during the manoeuvre. The vein has a cylindrical appearance with small longitudinal diameters (3 mm). Click here for file
